# Some characterizations of disjoint topological transitivity on Orlicz spaces

**DOI:** 10.1186/s13660-018-1681-3

**Published:** 2018-04-16

**Authors:** Chung-Chuan Chen, Wei-Shih Du

**Affiliations:** 1 Department of Mathematics Education, National Taichung University of Education, Taichung, Taiwan; 2 Department of Mathematics, National Kaohsiung Normal University, Kaohsiung, Taiwan

**Keywords:** 54H20, 46E30, 47A16, Topological transitivity, Disjoint topological transitivity, Weighted translation, Orlicz space, Locally compact group

## Abstract

In this paper, we study the disjointness of topological transitivity on Orlicz spaces endowed with the Orlicz norm. We first give the characterization for weighted translations to be topologically transitive. Based on this, a sufficient condition and a necessary condition for powers of weighted translations to be disjoint topologically transitive are obtained as well.

## Introduction

In [1, 2], we gave sufficient and necessary conditions for weighted translations to be topologically transitive and disjoint topologically transitive on the Lebesgue space of locally compact groups, which generalizes some results about weighted shifts on the discrete group $\mathbb{Z}$ in [3, 4], respectively. We note that the investigation of linear dynamics on locally compact groups has attracted a lot of attention. Indeed, let *G* be a locally compact group. The disjoint hypercyclicity of weighted translations on $L^{p}(G)$ was studied in [5]. The existence of transitive weighted translations on $L^{p}(G)$ is obtained in [6]. Also, Abakumov and Kuznetsova in [7] focused on the density of translates in the weighted Lebesgue space $L_{w}^{p}(G) $ where *w* is a weight on *G*. Recently, Azimi and Akbarbaglu in [8] extended the work of [2] from the Lebesgue space $L^{p}(G)$ to the Orlicz space $L^{\Phi}(G)$ endowed with the Luxemburg norm, where Φ is a Young function. It is well known that the Orlicz space is a generalization of the usual Lebesgue space. Inspired by these, we initial the study of linear dynamics in the setting of the Orlicz space $L^{\Phi}(G)$, but with respect to the Orlicz norm. Although the Orlicz norm and the Luxemburg norm are equivalent, they deduce the totally different descriptions of topological transitivity in [8, Theorem 2.3] and Theorem 2.1 in Sect. 2 by applying different approaches and methods. In fact, we also investigate deeper dynamical notions, namely, disjoint topological transitivity and disjoint topological mixing in Sect. 3, which were not studied in [8].

A bounded linear operator *T* on a separable Banach space *X* is said to be *topologically transitive* if, given two non-empty open sets $U,V\subset X$, there exists $n\in\mathbb{N}$ such that $T^{n}(U)\cap V\neq \emptyset$. If $T^{n}(U)\cap V\neq\emptyset$ from some *n* onwards, then *T* is *topologically mixing*. It should be noted [9] that topological transitivity and hypercyclicity are equivalent on separable Banach spaces. The latter notion arises from the invariant subset problem in analysis. An operator *T* is called *hypercyclic* if there is $x\in X$ such that its orbit under *T*, denoted by $Orb(T,x):=\{T^{n}x: n\in \mathbb{N}\} $, is dense in *X*. Hypercyclicity and linear dynamics have been studied intensely. We refer to these classic books [9–11] on this subject. About one decade ago, Bernal-González, Bès and Peris introduced new notions of linear dynamics, namely, disjoint topological transitivity and disjoint hypercyclicity in [12] and [3], respectively. Since then, disjoint topological transitivity and disjoint hypercyclicity were studied by many authors [11, 13–18]. These new notions, disjoint topological transitivity and disjoint hypercyclicity, are kinds of generalizations of topological transitivity and hypercyclicity, respectively. We first recall some definitions of disjointness [3, 12] for further discussion.

### Definition 1.1

Given $N\geq2$, the operators $T_{1},T_{2},\ldots,T_{N}$ acting on a separable Banach space *X* are *disjoint hypercyclic*, or *diagonally hypercyclic* (in short, *d-hypercyclic*) if there is some vector $(x,x,\ldots,x)$ in the diagonal of $X^{N}=X\times X\times \cdots\times X$ such that

$$ \bigl\{ (x,x,\ldots,x),(T_{1}x,T_{2}x,\ldots,T_{N}x), \bigl(T^{2}_{1}x,T^{2}_{2}x,\ldots,T^{2}_{N}x\bigr),\ldots\bigr\} $$

is dense in $X^{N}$; if this is the case, then we say $x\in X$ is a *d*-hypercyclic vector associated to the operators $T_{1},T_{2},\ldots,T_{N} $.

For topological dynamics, several new notions were given accordingly [3] as follows.

### Definition 1.2

Given $N\geq2$, the operators $T_{1},T_{2},\ldots,T_{N}$ on a separable Banach space *X* are *disjoint topologically transitive* or *diagonally topologically transitive* (in short, *d-topologically transitive*) if, given non-empty open sets $U,V_{1},\ldots,V_{N}\subset X$, there is some $n\in\mathbb{N}$ such that

$$ \emptyset\neq U\cap T_{1}^{-n}(V_{1})\cap T_{2}^{-n}(V_{2})\cap\cdots\cap T_{L}^{-n}(V_{N}). $$

If the above condition is satisfied from some *n* onwards, then $T_{1},T_{2},\ldots,T_{N}$ are called *disjoint topologically mixing* (in short, *d-topologically mixing*).

In this note, the characterizations for weighted translation operators on the Orlicz space to be topologically transitive and disjoint topologically transitive will be demonstrated. We introduce the Orlicz space briefly for further study. It should be noted the Orlicz space is defined by a Young function which highly relies on the Young inequality. Indeed, a continuous, even and convex function $\Phi:\mathbb{R}\rightarrow\mathbb{R}$ is called a *Young function* if it satisfies $\Phi(0)=0$, $\Phi(t)>0$ for $t>0$, and $\lim_{t\rightarrow\infty }\Phi(t)=\infty$. For a Young function Φ, the complementary function Ψ of Φ is given by

$$ \Psi(y)=\sup\bigl\{ x \vert y \vert-\Phi(x):x\geq0\bigr\} \quad (y\in \mathbb{R}), $$

which is also a Young function. If Ψ is the complementary function Φ, then Φ is the complementary function Ψ, and they both satisfy the Young inequality,

$$ xy\leq\Phi(x)+\Psi(y)\quad (x,y\geq0). $$

Let *G* be a locally compact group with identity *e* and a right Haar measure *λ*. Then the *Orlicz space* $L^{\Phi}(G)$ is defined by

$$ L^{\Phi}(G)= \biggl\{ f:G\rightarrow\mathbb{C}: \int_{G}\Phi\bigl(\alpha\vert f \vert\bigr)\,d\lambda< \infty\text{ for some $\alpha>0$} \biggr\} , $$

where *f* is a Borel measurable function. Moreover, the Orlicz space is a Banach space under the Orlicz norm defined for $f\in L^{\Phi}(G)$ by

$$ \Vert f \Vert _{\Phi}=\sup\biggl\{ \int_{G} \vert fv \vert\,d\lambda: \int_{G}\Psi\bigl( \vert v \vert\bigr)\,d\lambda\leq1 \biggr\} . $$

One can also define the Luxemburg norm on $L^{\Phi}(G)$ by

$$ N_{\Phi}(f)=\inf\biggl\{ k>0: \int_{G}\Phi\biggl(\frac{ \vert f \vert }{k} \biggr)\,d\lambda \leq1\biggr\} . $$

It is well known that these two norms above are equivalent.

We note that the Lebesgue space is a special case of the Orlicz space. The interesting properties and structures of Orlicz spaces have been investigated intensely over the last several decades. For instance, the properties $(T_{L^{\Phi}})$ and $(F_{L^{\Phi}})$ for Orlicz spaces $L^{\Phi}$ were studied by Tanaka in [19] recently. In [20], Piaggio also considered Orlicz spaces and the large scale geometry of Heintze groups. Hence it is significant to tackle hypercyclicity and linear dynamics on Orlicz spaces. For more discussion and recent work as regards Orlicz spaces, see [21–23].

We note that a Banach space admits a hypercyclic operator if, and only if, it is separable and infinite-dimensional [24, 25]. Hence we assume that *G* is second countable and Φ is $\Delta_{2}$-regular in this paper. A Young function is said to be $\Delta_{2}$-*regular* in [23] if there exist a constant $M>0$ and $t_{0}>0$ such that $\Phi(2t)\leq M\Phi(t)$ for $t\geq t_{0}$ when *G* is compact, and $\Phi(2t)\leq M\Phi(t)$ for all $t>0 $ when *G* is noncompact. For example, the Young functions Φ given by

$$ \Phi(t)=\frac{ \vert t \vert ^{p}}{p}\quad (1\leq p< \infty)\quad \mbox{and}\quad \Phi(t)= \vert t \vert^{\alpha}\bigl(1+ \bigl\vert \log\vert t \vert \bigr\vert \bigr)\quad (\alpha>1) $$

are both $\Delta_{2}$-regular in [8, 23]. If Φ is $\Delta_{2}$-regular, then the space $C_{c}(G)$ of all continuous functions on *G* with compact support is dense in $L^{\Phi}(G)$, and the dual space $(L^{\Phi}(G), \Vert \cdot \Vert _{\Phi})$ is $(L^{\Psi}(G),N_{\Psi}(\cdot))$.

A bounded continuous function $w:G \rightarrow(0,\infty)$ is called a *weight* on *G*. Let $a \in G$ and let $\delta_{a}$ be the unit point mass at *a*. A *weighted translation* on *G* is a weighted convolution operator $T_{a,w}: L^{\Phi}(G)\longrightarrow L^{\Phi}(G)$ defined by

$$ T_{a,w}(f)= wT_{a}(f)\quad \bigl(f \in L^{\Phi}(G)\bigr), $$

where *w* is a weight on *G* and $T_{a}(f)=f*\delta_{a}\in L^{\Phi}(G)$ is the convolution:

$$ (f*\delta_{a}) (x)= \int_{y\in G} f\bigl(xy^{-1}\bigr)\delta_{a}(y) = f\bigl(xa^{-1}\bigr)\quad (x\in G). $$

If $w^{-1}\in L^{\infty}(G)$, then we can define a self-map $S_{a,w}$ on $L^{\Phi}(G)$ by

$$ S_{a,w}(h) = \frac{h}{w}* \delta_{a^{-1}}\quad \bigl(h \in L^{\Phi}(G)\bigr) $$

so that

$$ T_{a,w}S_{a,w}(h) = h \quad\bigl(h \in L^{\Phi}(G)\bigr). $$

In what follows, we assume $w,w^{-1}\in L^{\infty}(G)$.

In Sect. 2, we will characterize topological transitivity of weighted translations on $L^{\Phi}(G)$ in terms of the weight, the Haar measure and the group element. Applying similar arguments, in Sect. 3, we will obtain a sufficient condition and a necessary condition for powers of weighted translations to be disjoint topologically transitive. In particular, the Minkowski inequality and some estimates with inequalities play important roles in demonstrating the results in the two sections.

## Topological transitivity

Now we are ready to give the result of topological transitivity. Applying the same argument, the characterization for topological mixing follows immediately.

### Theorem 2.1

*Let* *G* *be a locally compact group and let* $a\in G$. *Let* *w* *be a weight on* *G*, *and let* Φ *be a Young function*. *Let* $T_{a,w}$ *be a weighted translation on* $L^{\Phi}(G)$. *Then the following conditions are equivalent*.

(i) $T_{a,w}$ *is topologically transitive on* $L^{\Phi}(G)$.
(ii) *For each compact subset* *K* *of* *G* *with* $\lambda(K)>0$, *there exist a sequence of Borel sets* $(E_{k})$ *in* *K* *and a strictly increasing sequence* $(n_{k})\subset\mathbb{N}$ *such that*
  $$ \lim_{k \rightarrow\infty}\sup_{v\in\Omega} \int_{K\setminus{E_{k}}} \bigl\vert v(x) \bigr\vert \,d\lambda(x)=0, $$
  *and the two sequences*
  $$ \varphi_{n}:=\prod_{j=1}^{n}w \ast\delta_{a^{-1}}^{j}\quad\textit{and} \quad {\widetilde{\varphi}_{n}}:= \Biggl(\prod_{j=0}^{n-1}w \ast\delta_{a}^{j}\Biggr)^{-1} $$
  *satisfy*
  $$ \lim_{k \rightarrow\infty}\sup_{v\in \Omega} \int_{E_{k}}\varphi_{n_{k}}(x) \bigl\vert v \bigl(xa^{n_{k}}\bigr) \bigr\vert \,d\lambda(x)=0 $$
  *and*
  $$ \lim_{k \rightarrow\infty}\sup_{v\in\Omega} \int_{E_{k}}\widetilde{\varphi}_{n_{k}}(x) \bigl\vert v\bigl(xa^{-n_{k}}\bigr) \bigr\vert \,d\lambda(x)=0, $$
  *where* Ω *is the set of all Borel functions* *v* *on* *G* *satisfying* $\int_{G} \Psi( \vert v \vert )\,d\lambda\leq1$.

### Proof

(ii) ⇒ (i). Suppose that *U* and *V* are non-empty open subsets of $L^{\Phi}(G)$. Since the space $C_{c}(G)$ of all continuous functions on *G* with compact support is dense in $L^{\Phi}(G)$, there are $f,g\in C_{c}(G)$ such that $f\in U$ and $g\in V$. Let *K* be the union of supports of *f* and *g*. Given $\varepsilon>0$, by condition (ii), there exist $(E_{k})$ and $(n_{k})$ such that, for some *k*, we have

$$\begin{aligned} &\Vert f \Vert _{\infty}\cdot\sup_{v\in\Omega} \int_{K\setminus{E_{k}}} \bigl\vert v(x) \bigr\vert \,d\lambda(x)< \varepsilon, \\ &\Vert g \Vert _{\infty}\cdot\sup_{v\in\Omega} \int_{K\setminus{E_{k}}} \bigl\vert v(x) \bigr\vert \,d\lambda(x)< \varepsilon, \\ &\Vert f \Vert _{\infty}\cdot\sup_{v\in \Omega} \int_{E_{k}}\varphi_{n_{k}}(x) \bigl\vert v \bigl(xa^{n_{k}}\bigr) \bigr\vert \,d\lambda(x)< \varepsilon \end{aligned}$$

and

$$ \Vert g \Vert _{\infty}\cdot\sup_{v\in\Omega} \int_{E_{k}}\widetilde{\varphi}_{n_{k}}(x) \bigl\vert v\bigl(xa^{-n_{k}}\bigr) \bigr\vert \,d\lambda (x)< \varepsilon. $$

Therefore

$$\begin{aligned} \bigl\Vert T_{a,w}^{n_{k}}(f\chi_{E_{k}}) \bigr\Vert _{\Phi}&=\sup_{v\in\Omega}\int_{G} \bigl\vert T_{a,w}^{n_{k}}(f \chi_{E_{k}}) (x)v(x) \bigr\vert \,d\lambda(x) \\ &=\sup_{v\in\Omega} \int_{G} \bigl\vert w(x)w\bigl(xa^{-1}\bigr)\cdots w\bigl(xa^{-n_{k}+1}\bigr)f\bigl(xa^{-n_{k}}\bigr)\chi_{E_{k}} \bigl(xa^{-n_{k}}\bigr)v(x) \bigr\vert \,d\lambda(x) \\ &=\sup_{v\in\Omega} \int_{G} \bigl\vert w\bigl(xa^{n_{k}}\bigr)w \bigl(xa^{n_{k}-1}\bigr)\cdots w(xa)f(x)\chi_{E_{k}}(x)v \bigl(xa^{n_{k}}\bigr) \bigr\vert \,d\lambda(x) \\ &\leq \Vert f \Vert _{\infty}\cdot\sup_{v\in\Omega} \int_{E_{k}}\varphi_{n_{k}}(x) \bigl\vert v \bigl(xa^{n_{k}}\bigr) \bigr\vert \,d\lambda(x)< \varepsilon. \end{aligned}$$

Likewise,

$$\begin{aligned} \bigl\Vert S_{a,w}^{n_{k}}(g\chi_{E_{k}}) \bigr\Vert _{\Phi}&=\sup_{v\in\Omega}\int_{G} \bigl\vert S_{a,w}^{n_{k}}(g \chi_{E_{k}}) (x)v(x) \bigr\vert \,d\lambda(x) \\ &=\sup_{v\in\Omega} \int_{G}\frac{1}{ \vert w(xa)w(xa^{2})\cdots w(xa^{n_{k}}) \vert } \bigl\vert g\bigl(xa^{n_{k}} \bigr)\chi_{E_{k}}\bigl(xa^{n_{k}}\bigr)v(x) \bigr\vert \,d \lambda(x) \\ &=\sup_{v\in\Omega} \int_{G}\frac{1}{ \vert w(xa^{-n_{k}+1})w(xa^{-n_{k}+2})\cdots w(x) \vert } \bigl\vert g(x) \chi_{E_{k}}(x)v\bigl(xa^{-n_{k}}\bigr) \bigr\vert \,d\lambda(x) \\ &\leq \Vert g \Vert _{\infty}\cdot\sup_{v\in\Omega} \int_{E_{k}}\widetilde{\varphi}_{n_{k}}(x) \bigl\vert v\bigl(xa^{-n_{k}}\bigr) \bigr\vert \,d\lambda (x)< \varepsilon. \end{aligned}$$

Also,

$$\begin{aligned} \Vert f-f\chi_{E_{k}} \Vert _{\Phi}&=\sup _{v\in\Omega} \int_{G} \bigl\vert f(x)-f(x)\chi_{E_{k}}(x) \bigr\vert \bigl\vert v(x) \bigr\vert \,d\lambda(x) \\ &=\sup_{v\in\Omega} \int_{G} \bigl\vert f(x)\chi_{K\setminus E_{k}}(x) \bigr\vert \bigl\vert v(x) \bigr\vert \,d\lambda(x) \\ &=\sup_{v\in\Omega} \int_{K\setminus E_{k}} \bigl\vert f(x) \bigr\vert \bigl\vert v(x) \bigr\vert \,d\lambda(x) \\ &\leq \Vert f \Vert _{\infty}\cdot \int_{K\setminus E_{k}} \bigl\vert v(x) \bigr\vert \,d\lambda(x)< \varepsilon. \end{aligned}$$

Similarly,

$$ \Vert g-g\chi_{E_{k}} \Vert _{\Phi} \leq \Vert g \Vert _{\infty}\cdot \int_{K\setminus E_{k}} \bigl\vert v(x) \bigr\vert \,d\lambda(x)< \varepsilon. $$

Now let

$$ v_{k}=f\chi_{E_{k}}+S_{a,w}^{n_{k}}(g \chi_{E_{k}}). $$

Then, by the Minkowski inequality,

$$ \Vert v_{k}-f \Vert _{\Phi}\leq \Vert f \chi_{E_{k}}-f \Vert _{\Phi}+ \bigl\Vert S_{a,w}^{n_{k}}(g \chi_{E_{k}}) \bigr\Vert _{\Phi}< 2\varepsilon $$

and

$$ \bigl\Vert T_{a,w}^{n_{k}}v_{k}-g \bigr\Vert _{\Phi}\leq\bigl\Vert T_{a,w}^{n_{k}}(f \chi_{E_{k}}) \bigr\Vert _{\Phi}+ \Vert g\chi_{E_{k}}-g \Vert _{\Phi}< 2\varepsilon, $$

which implies

$$ T_{a,w}^{n_{k}}(U)\cap V\neq\emptyset $$

for some *k*. Therefore $T_{a,w}$ is topologically transitive.

(i) ⇒ (ii). Let $\varepsilon\in(0,1)$. Let *K* be a compact subset of *G*, and let $\chi_{K}$ be the characteristic function of *K*. By the assumption of topological transitivity, there exist $f\in L^{\Phi}(G)$ and some $m\in\mathbb{N}$ such that

$$ \Vert f-\chi_{K} \Vert _{\Phi} < \varepsilon^{2}\quad \mbox{and}\quad \bigl\Vert T_{a,w}^{m}f+\chi_{K} \bigr\Vert _{\Phi}< \varepsilon^{2}. $$

Without loss of generality, we may assume that *f* is real-valued by the continuity of the mapping $h\in L^{\Phi}(G,\mathbb{C})\mapsto \operatorname{Re} h\in L^{\Phi}(G,\mathbb{R})$ and the fact that $T_{a,w}$ commutes with it. Also, the mapping $h\in L^{\Phi}(G,\mathbb{R})\mapsto h^{+}\in L^{\Phi}(G,\mathbb{R})$ commutes with $T_{a,w}$ where $h^{+}=\max\{0,h\}$. Therefore, for a Borel set $F\subset G$, we have

$$\begin{aligned} \bigl\Vert \bigl(T_{a,w}^{m}f^{+}\bigr)\chi_{F} \bigr\Vert _{\Phi}&\leq \bigl\Vert \bigl(T_{a,w}^{m}f \bigr)^{+} \bigr\Vert _{\Phi}= \bigl\Vert \bigl(T_{a,w}^{m}f-(- \chi_{K})+(-\chi_{K})\bigr)^{+} \bigr\Vert _{\Phi}\\ &\leq \bigl\Vert \bigl(T_{a,w}^{m}f-(- \chi_{K})\bigr)^{+} \bigr\Vert _{\Phi}+ \bigl\Vert (- \chi_{K})^{+} \bigr\Vert _{\Phi}\\ &= \bigl\Vert \bigl(T_{a,w}^{m}f-(-\chi_{K}) \bigr)^{+} \bigr\Vert _{\Phi}\leq\bigl\Vert T_{a,w}^{m}f+ \chi_{K} \bigr\Vert _{\Phi}< \varepsilon^{2} \end{aligned}$$

and

$$\begin{aligned} \bigl\Vert f^{-}\chi_{F} \bigr\Vert _{\Phi}&\leq \bigl\Vert f^{-} \bigr\Vert _{\Phi}= \bigl\Vert (f-\chi_{K}+ \chi_{K})^{-} \bigr\Vert _{\Phi}\\ &\leq \bigl\Vert (f-\chi_{K})^{-} \bigr\Vert _{\Phi}+ \bigl\Vert \chi_{K}^{-} \bigr\Vert _{\Phi}\\ &= \Vert f-\chi_{K} \Vert _{\Phi}< \varepsilon^{2}, \end{aligned}$$

where $f^{-}=\max\{0,-f\}$.

Let $A=\{x\in K: \vert f(x)-1 \vert \geq\varepsilon\}$. Then

$$ f(x)>1-\varepsilon>0 \quad (x\in K\setminus A)\quad \mbox{and} \quad\sup_{v\in \Omega} \int_{A} \bigl\vert v(x) \bigr\vert \,d\lambda(x)< \varepsilon $$

by

$$\begin{aligned} \varepsilon^{2} &> \Vert f-\chi_{K} \Vert _{\Phi} \\ &= \sup_{v\in\Omega} \int_{G} \bigl\vert f(x)-\chi_{K}(x) \bigr\vert \bigl\vert v(x) \bigr\vert \,d\lambda(x) \\ &\geq \sup_{v\in\Omega} \int_{A} \bigl\vert f(x)-1 \bigr\vert \bigl\vert v(x) \bigr\vert \,d\lambda(x) \\ &> \sup_{v\in\Omega} \int_{A} \varepsilon\bigl\vert v(x) \bigr\vert \,d \lambda(x). \end{aligned}$$

Let $B_{m}=\{x\in K: \vert T_{a,w}^{m}f(x)+1 \vert \geq\varepsilon\} $. Then

$$ T_{a,w}^{m}f(x)< \varepsilon-1< 0\quad ( x\in K\setminus B_{m}) \quad \mbox{and}\quad \sup_{v\in\Omega} \int_{B_{m}} \bigl\vert v(x) \bigr\vert \,d\lambda(x)< \varepsilon $$

by the following estimate:

$$\begin{aligned} \varepsilon^{2} &> \bigl\Vert T_{a,w}^{m}f+ \chi_{K} \bigr\Vert _{\Phi} \\ &= \sup_{v\in\Omega} \int_{G} \bigl\vert T_{a,w}^{m}f(x)+ \chi_{K}(x) \bigr\vert \bigl\vert v(x) \bigr\vert \,d\lambda(x) \\ &\geq\sup_{v\in\Omega} \int_{B_{m}} \bigl\vert T_{a,w}^{m}f(x)+1 \bigr\vert \bigl\vert v(x) \bigr\vert \,d\lambda(x) \\ &> \sup_{v\in\Omega} \int_{B_{m}} \varepsilon\bigl\vert v(x) \bigr\vert \, d \lambda(x). \end{aligned}$$

Let $E_{m}=K\setminus(A\cup B_{m})$. Then

$$\begin{aligned} \varepsilon^{2} &> \bigl\Vert \bigl(T_{a,w}^{m}f^{+} \bigr)\chi_{E_{m}a^{m}} \bigr\Vert _{\Phi} \\ &= \sup_{v\in\Omega} \int_{E_{m}a^{m}} \bigl\vert T_{a,w}^{m}f^{+}(x) \bigr\vert \bigl\vert v(x) \bigr\vert \,d\lambda(x) \\ &= \sup_{v\in\Omega} \int_{E_{m}a^{m}} \bigl\vert w(x)w\bigl(xa^{-1}\bigr)\cdots w\bigl(xa^{-m+1}\bigr)f^{+}\bigl(xa^{-m}\bigr) \bigr\vert \bigl\vert v(x) \bigr\vert \,d\lambda(x) \\ &= \sup_{v\in\Omega} \int_{E_{m}} \bigl\vert w\bigl(xa^{m}\bigr)w \bigl(xa^{m-1}\bigr)\cdots w(xa)f^{+}(x) \bigr\vert \bigl\vert v \bigl(xa^{m}\bigr) \bigr\vert \,d\lambda(x) \\ &= \sup_{v\in\Omega} \int_{E_{m}}\varphi_{m}(x)f^{+}(x) \bigl\vert v \bigl(xa^{m}\bigr) \bigr\vert \,d\lambda(x) \\ &> \sup_{v\in \Omega} \int_{E_{m}}(1-\varepsilon)\varphi_{m}(x) \bigl\vert v\bigl(xa^{m}\bigr) \bigr\vert \,d\lambda(x). \end{aligned}$$

Hence

$$ \sup_{v\in\Omega} \int_{E_{m}}\varphi_{m}(x) \bigl\vert v \bigl(xa^{m}\bigr) \bigr\vert \,d\lambda(x)< \frac{\varepsilon^{2}}{1-\varepsilon}. $$

Similarly,

$$ \sup_{v\in\Omega} \int_{E_{m}}\widetilde{\varphi}_{m}(x) \bigl\vert v \bigl(xa^{-m}\bigr) \bigr\vert \,d\lambda(x)< \frac{\varepsilon^{2}}{1-\varepsilon} $$

by

$$\begin{aligned} \varepsilon^{2} &> \bigl\Vert f^{-}\chi_{E_{m}a^{-m}} \bigr\Vert _{\Phi} \\ &= \sup_{v\in \Omega} \int_{E_{m}a^{-m}} \bigl\vert S_{a,w}^{m} \bigl(T_{a,w}^{m}f^{-}\bigr) (x) \bigr\vert \bigl\vert v(x) \bigr\vert \,d\lambda(x) \\ &= \sup_{v\in\Omega} \int_{E_{m}a^{-m}} \bigl\vert w(xa)w\bigl(xa^{2}\bigr) \cdots w\bigl(xa^{m}\bigr) \bigl(T_{a,w}^{m}f \bigr)^{-}\bigl(xa^{m}\bigr) \bigr\vert \bigl\vert v(x) \bigr\vert \,d\lambda(x) \\ &= \sup_{v\in\Omega} \int_{E_{m}} \bigl\vert w\bigl(xa^{-m+1}\bigr)w \bigl(xa^{-m+2}\bigr)\cdots w(x) \bigl(T_{a,w}^{m}f \bigr)^{-}(x) \bigr\vert \bigl\vert v\bigl(xa^{-m}\bigr) \bigr\vert \,d\lambda(x) \\ &= \sup_{v\in\Omega} \int_{E_{m}}\widetilde{\varphi}_{m}(x) \bigl(T_{a}^{m}f\bigr)^{-}(x) \bigl\vert v \bigl(xa^{-m}\bigr) \bigr\vert \,d\lambda(x) \\ &> \sup_{v\in\Omega} \int_{E_{m}}(1-\varepsilon)\widetilde{\varphi}_{m}(x) \bigl\vert v\bigl(xa^{-m}\bigr) \bigr\vert \,d\lambda(x). \end{aligned}$$

Also, we have

$$\begin{aligned} & \sup_{v\in\Omega} \int_{K\setminus{E_{m}}} \bigl\vert v(x) \bigr\vert \,d\lambda(x) \\ &\quad= \sup_{v\in\Omega} \int_{A\cup B_{m}} \bigl\vert v(x) \bigr\vert \,d\lambda(x) \\ &\quad\leq \sup_{v\in\Omega} \int_{A} \bigl\vert v(x) \bigr\vert \,d\lambda(x) + \sup _{v\in \Omega} \int_{B_{m}} \bigl\vert v(x) \bigr\vert \,d\lambda(x) \\ &\quad< \varepsilon+\varepsilon=2\varepsilon. \end{aligned}$$

Combining all these, condition (ii) follows. □

Replacing the subsequence $(n_{k})$ by the full sequence $(n)$ in condition (ii) of Theorem 2.1, the characterization for $T_{a,w}$ to be topologically mixing on $L^{\Phi}(G)$ is obtained below.

### Corollary 2.2

*Let* *G* *be a locally compact group and let* $a\in G$. *Let* *w* *be a weight on* *G*, *and let* Φ *be a Young function*. *Let* $T_{a,w}$ *be a weighted translation on* $L^{\Phi}(G)$. *Then the following conditions are equivalent*.

(i) $T_{a,w}$ *is topologically mixing on* $L^{\Phi}(G)$.
(ii) *For each compact subset* *K* *of* *G* *with* $\lambda(K)>0$, *there exists a sequence of Borel sets* $(E_{n})$ *in* *K* *such that*
  $$ \lim_{n \rightarrow\infty}\sup_{v\in\Omega} \int_{K\setminus{E_{n}}} \bigl\vert v(x) \bigr\vert \,d\lambda(x)=0, $$
  *and the two sequences*
  $$ \varphi_{n}:=\prod_{j=1}^{n}w \ast\delta_{a^{-1}}^{j}\quad \textit{and} \quad {\widetilde{\varphi}_{n}}:= \Biggl(\prod_{j=0}^{n-1}w \ast\delta_{a}^{j}\Biggr)^{-1} $$
  *satisfy*
  $$ \lim_{n \rightarrow\infty}\sup_{v\in \Omega} \int_{E_{n}}\varphi_{n}(x) \bigl\vert v \bigl(xa^{n}\bigr) \bigr\vert \,d\lambda(x)=0 $$
  *and*
  $$ \lim_{n \rightarrow\infty}\sup_{v\in\Omega} \int_{E_{n}}\widetilde{\varphi}_{n}(x) \bigl\vert v\bigl(xa^{-n}\bigr) \bigr\vert \,d\lambda(x)=0, $$
  *where* Ω *is the set of all Borel functions* *v* *on* *G* *satisfying* $\int_{G} \Psi( \vert v \vert )\,d\lambda\leq1$.

### Proof

(ii) ⇒ (i). As in the proof of Theorem 2.1, let

$$ v_{n}=f\chi_{E_{n}}+S_{a,w}^{n}(g \chi_{E_{n}}). $$

Then

$$ \Vert v_{n}-f \Vert _{\Phi}\rightarrow0 \quad\mbox{and}\quad \bigl\Vert T_{a,w}^{n}v_{n}-g \bigr\Vert _{\Phi}\rightarrow0 $$

as $n\rightarrow\infty$. Hence $T_{a,w}$ is topologically mixing.

(i) ⇒ (ii). Let $\varepsilon\in(0,1)$. Let *K* be a compact subset of *G*, and let $\chi_{K}$ be the characteristic function of *K*. By the assumption of topological mixing, there exists $f\in L^{\Phi}(G)$ such that

$$ \Vert f-\chi_{K} \Vert _{\Phi} < \varepsilon^{2}\quad \mbox{and}\quad \bigl\Vert T_{a,w}^{n}f+\chi_{K} \bigr\Vert _{\Phi}< \varepsilon^{2}. $$

Applying a similar argument as in the proof of Theorem 2.1, one can obtain condition (ii). □

## Disjoint topological transitivity

In this section, we turn our attention to give sufficient conditions and necessary conditions for powers of weighted translations on $L^{\Phi}(G)$ to be disjoint topologically transitive and disjoint topologically mixing.

### Theorem 3.1

*Let* *G* *be a locally compact group*, *and let* $a\in G$. *Let* Φ *be a Young function*. *Given some* $N\geq2$, *let* $T_{l}=T_{a,w_{l}}$ *be a weighted translation on* $L^{\Phi}(G)$, *generated by a weight* $w_{l}$ *on* *G* *for* $1\leq l \leq N$. *Let* $r_{l}\in\mathbb{N}$ *for* $1\leq l \leq N$. *For* $1\leq r_{1}< r_{2}< \cdots< r_{N}$, (ii) *implies* (i).

(i) $T_{1}^{r_{1}}, T_{2}^{r_{2}},\ldots, T_{N}^{r_{N}}$ *are disjoint topologically transitive on* $L^{\Phi}(G)$.
(ii) *For each compact subset* *K* *of* *G* *with* $\lambda(K)>0$, *there exist a sequence of Borel sets* $(E_{k})$ *in* *K* *and a strictly increasing sequence* $(n_{k})\subset\mathbb{N}$ *such that*
  $$ \lim_{k \rightarrow\infty}\sup_{v\in\Omega} \int_{K\setminus{E_{k}}} \bigl\vert v(x) \bigr\vert \,d\lambda(x)=0, $$
  *and the two sequences*
  $$ \varphi_{l,r_{l}n}:=\prod_{j=1}^{r_{l}n}w_{l} \ast\delta_{a^{-1}}^{j}\quad \textit{and} \quad{\widetilde{\varphi}_{l,r_{l}n}}:= \Biggl(\prod_{j=0}^{r_{l}n-1}w_{l} \ast \delta_{a}^{j} \Biggr)^{-1} $$
  *satisfy* (*for* $1\leq l \leq N$)
  $$\begin{aligned} &\lim_{k \rightarrow\infty}\sup_{v\in \Omega} \int_{E_{k}}\varphi_{l,r_{l}n_{k}}(x) \bigl\vert v \bigl(xa^{r_{l}n_{k}}\bigr) \bigr\vert \,d\lambda(x)=0, \\ &\lim_{k \rightarrow\infty}\sup_{v\in\Omega} \int_{E_{k}}\widetilde{\varphi}_{l,r_{l}n_{k}}(x) \bigl\vert v\bigl(xa^{-r_{l}n_{k}}\bigr) \bigr\vert \,d\lambda(x)=0, \end{aligned}$$
  *and* (*for* $1\leq s < l \leq L$)
  $$\begin{aligned} &\lim_{k \rightarrow\infty}\sup_{v\in\Omega} \int_{E_{k}}\frac{{\widetilde{\varphi}_{s,(r_{l}-r_{s})n_{k}}}\cdot{\widetilde{\varphi}_{l,r_{l}n_{k}}}}{{ \widetilde{\varphi}_{s,r_{l}n_{k}}}}(x) \bigl\vert v \bigl(xa^{r_{s}n_{k}-r_{l}n_{k}}\bigr) \bigr\vert \,d\lambda(x)=0, \\ &\lim_{k \rightarrow\infty}\sup_{v\in\Omega} \int_{E_{k}}\frac{\varphi_{l,(r_{l}-r_{s})n_{k}}\cdot{\widetilde{\varphi}_{s,r_{s}n_{k}}}}{{\widetilde{\varphi}_{l,r_{s}n_{k}}}}(x) \bigl\vert v \bigl(xa^{r_{l}n_{k}-r_{s}n_{k}}\bigr) \bigr\vert \,d\lambda(x)=0, \end{aligned}$$
  *where* Ω *is the set of all Borel functions* *v* *on* *G* *satisfying* $\int_{G} \Psi( \vert v \vert )\,d\lambda\leq1$.

### Proof

We show that $T_{1}^{r_{1}}, T_{2}^{r_{2}},\ldots, T_{N}^{r_{N}}$ are disjoint topologically transitive. For $1\leq l\leq N$, let *U* and $V_{l}$ be non-empty open subsets of $L^{\Phi}(G)$. Since the space $C_{c}(G)$ of continuous functions on *G* with compact support is dense in $L^{\Phi}(G)$, we can pick $f,g_{l}\in C_{c}(G)$ with $f\in U$ and $g_{l}\in V_{l}$ for $l=1,2,\ldots,N$. Let *K* be the union of the compact supports of *f* and all $g_{l}$. Let $E_{k}\subset K$ and the sequences $(\varphi _{l,r_{l}n_{k}}), ({\widetilde{\varphi}_{l,r_{l}n_{k}}})$ satisfy condition (ii).

First, for $1\leq l\leq N$, we have

$$\begin{aligned} &\bigl\Vert T_{l}^{r_{l}n_{k}}(f\chi_{E_{k}}) \bigr\Vert _{\Phi} \\ &\quad=\sup_{v\in\Omega} \int_{G} \bigl\vert T_{l}^{r_{l}n_{k}}(f \chi_{E_{k}}) (x)v(x) \bigr\vert \,d\lambda(x) \\ &\quad=\sup_{v\in\Omega} \int_{G} \bigl\vert w_{l}(x)w_{l} \bigl(xa^{-1}\bigr)\cdots w_{l}\bigl(xa^{-r_{l}n_{k}+1}\bigr)f \bigl(xa^{-r_{l}n_{k}}\bigr)\chi_{E_{k}}\bigl(xa^{-r_{l}n_{k}}\bigr)v(x) \bigr\vert \,d\lambda(x) \\ &\quad=\sup_{v\in\Omega} \int_{G} \bigl\vert w_{l}\bigl(xa^{r_{l}n_{k}} \bigr)w_{l}\bigl(xa^{r_{l}n_{k}-1}\bigr)\cdots w_{l}(xa)f(x) \chi_{E_{k}}(x)v\bigl(xa^{r_{l}n_{k}}\bigr) \bigr\vert \,d\lambda(x) \\ &\quad \leq \Vert f \Vert _{\infty}\cdot\sup_{v\in\Omega} \int_{E_{k}}\varphi_{l,r_{l}n_{k}}(x) \bigl\vert v \bigl(xa^{r_{l}n_{k}}\bigr) \bigr\vert \,d\lambda(x)\rightarrow0 \end{aligned}$$

as $k\longrightarrow\infty$. Here we denote $S_{a,w_{l}}$ by $S_{l}$. Applying similar arguments to the iterates $S_{l}^{r_{l}n_{k}}$, and using the sequence $(\widetilde{\varphi}_{l,r_{l}n_{k}})$, for $1\leq l\leq N$, we have

$$\begin{aligned} & \bigl\Vert S_{l}^{r_{l}n_{k}}(g_{l} \chi_{E_{k}}) \bigr\Vert _{\Phi} \\ &\quad=\sup_{v\in\Omega} \int_{G} \bigl\vert S_{l}^{r_{l}n_{k}}(g_{l} \chi_{E_{k}}) (x)v(x) \bigr\vert \,d\lambda(x) \\ &\quad=\sup_{v\in\Omega} \int_{G}\frac{1}{ \vert w_{l}(xa)w_{l}(xa^{2})\cdots w_{l}(xa^{r_{l}n_{k}}) \vert } \bigl\vert g_{l} \bigl(xa^{r_{l}n_{k}}\bigr)\chi_{E_{k}}\bigl(xa^{r_{l}n_{k}}\bigr)v(x) \bigr\vert \,d\lambda(x) \\ &\quad=\sup_{v\in\Omega} \int_{G}\frac{1}{ \vert w_{l}(xa^{-r_{l}n_{k}+1})w_{l}(xa^{-r_{l}n_{k}+2})\cdots w_{l}(x) \vert } \bigl\vert g_{l}(x) \chi_{E_{k}}(x)v\bigl(xa^{-r_{l}n_{k}}\bigr) \bigr\vert \,d\lambda(x) \\ &\quad\leq \Vert g_{l} \Vert _{\infty}\cdot\sup _{v\in\Omega} \int_{E_{k}}\widetilde{\varphi}_{l,r_{l}n_{k}}(x) \bigl\vert v\bigl(xa^{-r_{l}n_{k}}\bigr) \bigr\vert \,d\lambda (x)\rightarrow0 \end{aligned}$$

as $k\longrightarrow\infty$. Moreover, for $1\leq s< l\leq N$, we have

$$\begin{aligned} & \bigl\Vert T_{l}^{r_{l}n_{k}}\bigl(S_{s}^{r_{s}n_{k}}g_{s} \chi_{E_{k}}\bigr) \bigr\Vert _{\Phi}\\ &\quad=\sup_{v\in\Omega} \int_{G} \bigl\vert w_{l}(x)w_{l} \bigl(xa^{-1}\bigr)\cdots w_{l}\bigl(xa^{-(r_{l}n_{k}-1)}\bigr) \bigr\vert \bigl\vert S_{s}^{r_{s}n_{k}}g_{s} \chi_{E_{k}}\bigl(xa^{-r_{l}n_{k}}\bigr)v(x) \bigr\vert \,d\lambda(x) \\ &\quad=\sup_{v\in\Omega} \int_{G}\frac{ \vert w_{l}(x)w_{l}(xa^{-1})\cdots w_{l}(xa^{-(r_{l}n_{k}-1)}) \vert }{ \vert w_{s}(xa^{-r_{l}n_{k}+1})w_{s}(xa^{-r_{l}n_{k}+2})\cdots w_{s}(xa^{-r_{l}n_{k}+r_{s}n_{k}}) \vert } \\ & \qquad{}\times\bigl\vert g_{s}\chi_{E_{k}}\bigl(xa^{-r_{l}n_{k}+r_{s}n_{k}} \bigr)v(x) \bigr\vert \,d\lambda(x) \\ &\quad=\sup_{v\in\Omega} \int_{E_{k}}\frac{\vert w_{l}(xa^{-(r_{s}-r_{l})n_{k}})w_{l}(xa^{-(r_{s}-r_{l})n_{k}-1})\cdots w_{l}(xa^{-(r_{s}n_{k}-1)}) \vert }{ \vert w_{s}(xa^{-(r_{s}n_{k}-1)})w_{s}(xa^{-(r_{s}n_{k}-2)})\cdots w_{s}(x) \vert ^{p}}\\ &\qquad{}\times \bigl\vert g_{s}(x)v \bigl(xa^{r_{l}n_{k}-r_{s}n_{k}}\bigr) \bigr\vert \,d\lambda(x) \\ &\quad=\sup_{v\in\Omega} \int_{E_{k}}\frac{\varphi_{l,(r_{l}-r_{s})n_{k}}(x)\cdot{\widetilde{\varphi}_{s,r_{s}n_{k}}}(x)}{{\widetilde{\varphi}_{l,r_{s}n_{k}}}(x)}\bigl\vert g_{s}(x)v\bigl(xa^{r_{l}n_{k}-r_{s}n_{k}}\bigr) \bigr\vert \,d\lambda(x) \\ &\quad \leq \Vert g_{s} \Vert _{\infty}\cdot\sup _{v\in\Omega} \int_{E_{k}}\frac{\varphi_{l,(r_{l}-r_{s})n_{k}}(x)\cdot{\widetilde{\varphi}_{s,r_{s}n_{k}}}(x)}{{\widetilde{\varphi}_{l,r_{s}n_{k}}}(x)} \bigl\vert v \bigl(xa^{r_{l}n_{k}-r_{s}n_{k}}\bigr) \bigr\vert \,d\lambda(x)\longrightarrow0 \end{aligned}$$

as $k\longrightarrow\infty$. Similarly, we have

$$\begin{aligned} & \bigl\Vert T_{s}^{r_{s}n_{k}}\bigl(S_{l}^{r_{l}n_{k}}g_{l} \chi_{E_{k}}\bigr) \bigr\Vert _{\Phi}\\ &\quad=\sup_{v\in\Omega} \int_{G} \bigl\vert w_{s}(x)w_{s} \bigl(xa^{-1}\bigr)\cdots w_{s}\bigl(xa^{-(r_{s}n_{k}-1)}\bigr) \bigr\vert \bigl\vert S_{l}^{r_{l}n_{k}}g_{l} \chi_{E_{k}}\bigl(xa^{-r_{s}n_{k}}\bigr)v(x) \bigr\vert \,d\lambda(x) \\ &\quad=\sup_{v\in\Omega} \int_{G}\frac{ \vert w_{s}(x)w_{s}(xa^{-1})\cdots w_{s}(xa^{-(r_{s}n_{k}-1)}) \vert }{ \vert w_{l}(xa^{-r_{s}n_{k}+1})w_{l}(xa^{-r_{s}n_{k}+2})\cdots w_{l}(xa^{-r_{s}n_{k}+r_{l}n_{k}}) \vert } \\ &\qquad{}\times \bigl\vert g_{l}\chi_{E_{k}}\bigl(xa^{-r_{s}n_{k}+r_{l}n_{k}} \bigr)v(x) \bigr\vert \,d\lambda(x) \\ &\quad=\sup_{v\in\Omega} \int_{E_{k}}\frac{\vert w_{s}(xa^{-(r_{l}-r_{s})n_{k}})w_{s}(xa^{-(r_{l}-r_{s})n_{k}-1})\cdots w_{s}(xa^{-(r_{l}n_{k}-1)}) \vert }{ \vert w_{l}(xa^{-(r_{l}n_{k}-1)})w_{l}(xa^{-(r_{l}n_{k}-2)})\cdots w_{l}(x) \vert }\\ &\qquad{}\times \bigl\vert g_{l}(x)v \bigl(xa^{-(r_{l}n_{k}-r_{s}n_{k})}\bigr) \bigr\vert \,d\lambda(x) \\ &\quad=\sup_{v\in\Omega} \int_{E_{k}}\frac{\widetilde{\varphi}_{l,(r_{l}-r_{s})n_{k}}(x)\cdot{\widetilde{\varphi}_{l,r_{l}n_{k}}}(x)}{{\widetilde{\varphi}_{s,r_{l}n_{k}}}(x)}\bigl\vert g_{l}(x)v\bigl(xa^{-(r_{l}n_{k}-r_{s}n_{k})}\bigr) \bigr\vert \,d\lambda(x) \\ &\quad\leq \Vert g_{l} \Vert _{\infty}\cdot\sup _{v\in\Omega} \int_{E_{k}}\frac{\widetilde{\varphi}_{s,(r_{l}-r_{s})n_{k}}(x)\cdot{\widetilde{\varphi}_{l,r_{l}n_{k}}}(x)}{{\widetilde{\varphi}_{s,r_{l}n_{k}}}(x)} \bigl\vert v \bigl(xa^{-(r_{l}n_{k}-r_{s}n_{k})}\bigr) \bigr\vert \,d\lambda (x)\longrightarrow0 \end{aligned}$$

as $k\longrightarrow\infty$. Also, as in the proof of Theorem 2.1, we have

$$ \lim_{k\rightarrow\infty} \Vert f\chi_{E_{k}}-f \Vert _{\Phi}=\lim_{k\rightarrow\infty} \Vert g_{l} \chi_{E_{k}}-g_{l} \Vert _{\Phi}=0 $$

by the condition

$$ \lim_{k \rightarrow\infty}\sup_{v\in\Omega} \int_{K\setminus{E_{k}}} \bigl\vert v(x) \bigr\vert \,d\lambda(x)=0. $$

Now for each $k\in\mathbb{N}$, we let

$$ v_{k}=f\chi_{E_{k}}+S^{r_{1}n_{k}}_{1}(g_{1} \chi_{E_{k}})+S^{r_{2}n_{k}}_{2}(g_{2} \chi_{E_{k}})+\cdots+S^{r_{N}n_{k}}_{N}(g_{N} \chi_{E_{k}}). $$

Then, by using the Minkowski inequality, we arrive at

$$ \Vert v_{k}-f \Vert _{\Phi}\leq \Vert f \chi_{E_{k}}-f \Vert _{\Phi}+\sum_{l=1}^{N} \bigl\Vert S^{r_{l}n_{k}}_{l}(g_{l} \chi_{E_{k}}) \bigr\Vert _{\Phi}$$

and

$$\begin{aligned} & \bigl\Vert T^{r_{l}n_{k}}_{l}v_{k}-g_{l} \bigr\Vert _{\Phi}\\ &\quad\leq \bigl\Vert T^{r_{l}n_{k}}_{l}(f\chi_{E_{k}}) \bigr\Vert _{\Phi}+ \bigl\Vert T^{r_{l}n_{k}}_{l}S_{1}^{r_{1}n_{k}}(g_{1}\chi_{E_{k}}) \bigr\Vert _{\Phi}+\cdots+ \bigl\Vert T^{r_{l}n_{k}}_{l}S_{l-1}^{r_{l-1}n_{k}}(g_{l-1} \chi_{E_{k}}) \bigr\Vert _{\Phi}\\ &\qquad{}+ \Vert g_{l}\chi_{E_{k}}-g_{l} \Vert _{\Phi}+ \bigl\Vert T^{r_{l}n_{k}}_{l}S_{l+1}^{r_{l+1}n_{k}}(g_{l+1}\chi_{E_{k}}) \bigr\Vert _{\Phi}+\cdots+ \bigl\Vert T^{r_{l}n_{k}}_{l}S_{N}^{r_{N}n_{k}}(g_{N} \chi_{E_{k}}) \bigr\Vert _{\Phi}. \end{aligned}$$

Hence $\lim_{k\rightarrow\infty}v_{k}=f$ and $\lim_{k\rightarrow \infty}T_{l}^{r_{l}n_{k}}v_{k}=g_{l}$ for $l=1,2,\ldots,N$, which implies

$$ \emptyset\neq U\cap T_{1}^{-{r_{1}n_{k}}}(V_{1})\cap T_{2}^{-{r_{2}n_{k}}}(V_{2})\cap\cdots\cap T_{N}^{-{r_{N}n_{k}}}(V_{N}). $$

 □

As in Sect. 2, one can strengthen the weight condition to characterize disjoint topological mixing.

### Corollary 3.2

*Let* *G* *be a locally compact group*, *and let* $a\in G$. *Let* Φ *be a Young function*. *Given some* $N\geq2$, *let* $T_{l}=T_{a,w_{l}}$ *be a weighted translation on* $L^{\Phi}(G)$, *generated by a weight* $w_{l}$ *on* *G* *for* $1\leq l\leq N$. *Let* $r_{l}\in\mathbb{N}$ *for* $1\leq l \leq N$. *For* $1\leq r_{1}< r_{2}< \cdots< r_{N}$, *we have* (ii) *implies* (i).

(i) $T_{1}^{r_{1}}, T_{2}^{r_{2}},\ldots, T_{N}^{r_{N}}$ *are disjoint topologically mixing on* $L^{\Phi}(G)$.
(ii) *For each compact subset* *K* *of* *G* *with* $\lambda(K)>0$, *there exists a sequence of Borel sets* $(E_{n})$ *in* *K* *such that*
  $$ \lim_{n \rightarrow\infty}\sup_{v\in\Omega} \int_{K\setminus{E_{n}}} \bigl\vert v(x) \bigr\vert \,d\lambda(x)=0, $$
  *and the two sequences*
  $$ \varphi_{l,r_{l}n}:=\prod_{j=1}^{r_{l}n}w_{l} \ast\delta_{a^{-1}}^{j}\quad \textit{and} \quad {\widetilde{\varphi}_{l,r_{l}n}}:= \Biggl(\prod_{j=0}^{r_{l}n-1}w_{l} \ast \delta_{a}^{j} \Biggr)^{-1} $$
  *satisfy* (*for* $1\leq l \leq N$)
  $$\begin{aligned} &\lim_{n \rightarrow\infty}\sup_{v\in \Omega} \int_{E_{n}}\varphi_{l,r_{l}n}(x) \bigl\vert v \bigl(xa^{r_{l}n}\bigr) \bigr\vert \,d\lambda(x)=0, \\ &\lim_{n \rightarrow\infty}\sup_{v\in\Omega} \int_{E_{n}}\widetilde{\varphi}_{l,r_{l}n}(x) \bigl\vert v\bigl(xa^{-r_{l}n}\bigr) \bigr\vert \,d\lambda(x)=0, \end{aligned}$$
  *and* (*for* $1\leq s < l \leq L$)
  $$\begin{aligned} &\lim_{n \rightarrow\infty}\sup_{v\in\Omega} \int_{E_{n}}\frac{{\widetilde{\varphi}_{s,(r_{l}-r_{s})n}}\cdot{\widetilde{\varphi}_{l,r_{l}n}}}{{\widetilde{\varphi}_{s,r_{l}n}}}(x) \bigl\vert v \bigl(xa^{r_{s}n-r_{l}n}\bigr) \bigr\vert \,d\lambda(x)=0, \\ &\lim_{n\rightarrow\infty}\sup_{v\in\Omega} \int_{E_{n}}\frac{\varphi_{l,(r_{l}-r_{s})n}\cdot{\widetilde{\varphi}_{s,r_{s}n}}}{{\widetilde{\varphi}_{l,r_{s}n}}}(x) \bigl\vert v \bigl(xa^{r_{l}n-r_{s}n}\bigr) \bigr\vert \,d\lambda(x)=0, \end{aligned}$$
  *where* Ω *is the set of all Borel functions* *v* *on* *G* *satisfying* $\int_{G} \Psi( \vert v \vert )\,d\lambda\leq1$.

Apart from the above sufficient condition for disjoint topological transitivity, one can deduce a necessary condition below.

### Theorem 3.3

*Let* *G* *be a locally compact group*, *and let* $a\in G$. *Let* Φ *be a Young function*. *Given some* $N\geq2$, *let* $T_{l}=T_{a,w_{l}}$ *be a weighted translation on* $L^{\Phi}(G)$, *generated by a weight* $w_{l}$ *on* *G* *for* $1\leq l \leq N$. *Let* $r_{l}\in\mathbb{N}$ *for* $1\leq l \leq N$. *For* $1\leq r_{1}< r_{2}< \cdots< r_{N}$, *we have* (i) *implies* (ii).

(i) $T_{1}^{r_{1}}, T_{2}^{r_{2}},\ldots, T_{N}^{r_{N}}$ *are disjoint topologically transitive on* $L^{\Phi}(G)$.
(ii) *For each compact subset* *K* *of* *G* *with* $\lambda(K)>0$, *there exist a sequence of Borel sets* $(E_{k})$ *in* *K* *and a strictly increasing sequence* $(n_{k})\subset\mathbb{N}$ *such that*
  $$ \lim_{k \rightarrow\infty}\sup_{v\in\Omega} \int_{K\setminus{E_{k}}} \bigl\vert v(x) \bigr\vert \,d\lambda(x)=0, $$
  *and the two sequences*
  $$ \varphi_{l,r_{l}n}:=\prod_{j=1}^{r_{l}n}w_{l} \ast\delta_{a^{-1}}^{j}\quad \textit{and}\quad {\widetilde{\varphi}_{l,r_{l}n}}:= \Biggl(\prod_{j=0}^{r_{l}n-1}w_{l} \ast \delta_{a}^{j} \Biggr)^{-1} $$
  *satisfy* (*for* $1\leq l \leq N$)
  $$\begin{aligned} &\lim_{k \rightarrow\infty}\sup_{v\in \Omega} \int_{E_{k}}\varphi_{l,r_{l}n_{k}}(x) \bigl\vert v \bigl(xa^{r_{l}n_{k}}\bigr) \bigr\vert \,d\lambda(x)=0, \\ &\lim_{k \rightarrow\infty}\sup_{v\in\Omega} \int_{E_{k}}\widetilde{\varphi}_{l,r_{l}n_{k}}(x) \bigl\vert v\bigl(xa^{-r_{l}n_{k}}\bigr) \bigr\vert \,d\lambda(x)=0, \end{aligned}$$
  *where* Ω *is the set of all Borel functions* *v* *on* *G* *satisfying* $\int_{G} \Psi( \vert v \vert )\,d\lambda\leq1$.

### Proof

Let $T_{1}^{r_{1}}, T_{2}^{r_{2}},\ldots, T_{N}^{r_{N}}$ be disjoint transitive. Let $\varepsilon\in(0,1)$, and let $K\subset G$ be a compact set with $\lambda(K)>0$. Then there exist $f\in L^{\Phi}(G)$ and some $m\in \mathbb{N}$ such that

$$ \Vert f-\chi_{K} \Vert _{\Phi} < \varepsilon^{2}\quad \mbox{and}\quad \bigl\Vert T_{l}^{r_{l}m}f+\chi_{K} \bigr\Vert _{\Phi}< \varepsilon^{2}. $$

Let

$$ A=\bigl\{ x\in K: \bigl\vert f(x)-1 \bigr\vert \geq\varepsilon\bigr\} \quad \mbox{and} \quad B_{l,m}=\bigl\{ x\in K: \bigl\vert T_{l}^{r_{l}m}f(x)+1 \bigr\vert \geq\varepsilon\bigr\} . $$

Let

$$ E_{k}=(K\setminus A)\setminus\bigcup_{1\leq l\leq N}B_{l,m}. $$

Then as in the proof of Theorem 2.1, one can obtain

$$ \sup_{v\in\Omega} \int_{E_{k}}\varphi_{l,r_{l}m}(x) \bigl\vert v \bigl(xa^{r_{l}m}\bigr) \bigr\vert \,d\lambda(x)< \frac{\varepsilon^{2}}{1-\varepsilon} $$

and

$$ \sup_{v\in\Omega} \int_{E_{k}}\widetilde{\varphi}_{l,r_{l}m}(x) \bigl\vert v \bigl(xa^{-r_{l}m}\bigr) \bigr\vert \,d\lambda(x)< \frac{\varepsilon^{2}}{1-\varepsilon}. $$

Also, as in the proof of Theorem 2.1,

$$ \sup_{v\in\Omega} \int_{A} \bigl\vert v(x) \bigr\vert \,d\lambda(x)< \varepsilon\quad\mbox{and}\quad \sup_{v\in\Omega} \int_{B_{l,m}} \bigl\vert v(x) \bigr\vert \,d\lambda(x)< \varepsilon. $$

Hence

$$\begin{aligned} &\sup_{v\in\Omega} \int_{K\setminus{E_{m}}} \bigl\vert v(x) \bigr\vert \,d\lambda(x) \\ &\quad= \sup_{v\in\Omega} \int_{A\cup B_{1,m} \cup\cdots\cup B_{N,m} } \bigl\vert v(x) \bigr\vert \,d\lambda(x) \\ &\quad\leq \sup_{v\in\Omega} \int_{A} \bigl\vert v(x) \bigr\vert \,d\lambda(x) + \sum _{l=1}^{N} \sup_{v\in \Omega} \int_{B_{l,m}} \bigl\vert v(x) \bigr\vert \,d\lambda(x) \\ &\quad < \varepsilon+N \varepsilon=(N+1)\varepsilon. \end{aligned}$$

Combining all these, condition (ii) follows. □

Similarly, a necessary condition for disjoint topological mixing follows.

### Corollary 3.4

*Let* *G* *be a locally compact group*, *and let* $a\in G$. *Let* Φ *be a Young function*. *Given some* $N\geq2$, *let* $T_{l}=T_{a,w_{l}}$ *be a weighted translation on* $L^{\Phi}(G)$, *generated by a weight* $w_{l}$ *on* *G* *for* $1\leq l \leq N$. *Let* $r_{l}\in\mathbb{N}$ *for* $1\leq l \leq N$. *For* $1\leq r_{1}< r_{2}< \cdots< r_{N}$, *we have* (i) *implies* (ii).

(i) $T_{1}^{r_{1}}, T_{2}^{r_{2}},\ldots, T_{N}^{r_{N}}$ *are disjoint topologically mixing on* $L^{\Phi}(G)$.
(ii) *For each compact subset* *K* *of* *G* *with* $\lambda(K)>0$, *there exists a sequence of Borel sets* $(E_{n})$ *in* *K* *such that*
  $$ \lim_{n \rightarrow\infty}\sup_{v\in\Omega} \int_{K\setminus{E_{n}}} \bigl\vert v(x) \bigr\vert \,d\lambda(x)=0, $$
  *and the two sequences*
  $$ \varphi_{l,r_{l}n}:=\prod_{j=1}^{r_{l}n}w_{l} \ast\delta_{a^{-1}}^{j}\quad \textit{and} \quad{\widetilde{\varphi}_{l,r_{l}n}}:= \Biggl(\prod_{j=0}^{r_{l}n-1}w_{l} \ast \delta_{a}^{j} \Biggr)^{-1} $$
  *satisfy* (*for* $1\leq l \leq N$)
  $$\begin{aligned} &\lim_{n \rightarrow\infty}\sup_{v\in \Omega} \int_{E_{n}}\varphi_{l,r_{l}n}(x) \bigl\vert v \bigl(xa^{r_{l}n}\bigr) \bigr\vert \,d\lambda(x)=0, \\ &\lim_{n \rightarrow\infty}\sup_{v\in\Omega} \int_{E_{n}}\widetilde{\varphi}_{l,r_{l}n}(x) \bigl\vert v\bigl(xa^{-r_{l}n}\bigr) \bigr\vert \,d\lambda(x)=0, \end{aligned}$$
  *where* Ω *is the set of all Borel functions* *v* *on* *G* *satisfying* $\int_{G} \Psi( \vert v \vert )\,d\lambda\leq1$.

## References

[1] Chen C.-C. (2017). Disjoint hypercyclic weighted translations on groups. Banach J. Math. Anal..

[2] Chen C.-C., Chu C.-H. (2011). Hypercyclic weighted translations on groups. Proc. Am. Math. Soc..

[3] Bès J., Peris A. (2007). Disjointness in hypercyclicity. J. Math. Anal. Appl..

[4] Salas H. (1995). Hypercyclic weighted shifts. Trans. Am. Math. Soc..

[5] Zhang L., Lu H.-Q., Fu X.-M., Zhou Z.-H. (2017). Disjoint hypercyclic powers of weighted translations on groups. Czechoslov. Math. J..

[6] Chen K.-Y. (2018). On aperiodicity and hypercyclic weighted translation operators. J. Math. Anal. Appl..

[7] Abakumov E., Kuznetsova Y. (2017). Density of translates in weighted $L^{p}$ spaces on locally compact groups. Monatshefte Math..

[8] Azimi M.R., Akbarbaglu I. (2018). Hypercyclicity of weighted translations on Orlicz spaces. Oper. Matrices.

[9] Grosse-Erdmann K.-G., Peris A. (2011). Linear Chaos.

[10] Bayart F., Matheron É. (2009). Dynamics of Linear Operators.

[11] Kostić M. (2015). Abstract Volterra Integro-Differential Equations.

[12] Bernal-González L. (2007). Disjoint hypercyclic operators. Stud. Math..

[13] Bès J., Martin Ö., Peris A., Shkarin S. (2012). Disjoint mixing operators. J. Funct. Anal..

[14] Bès J., Martin Ö., Sanders R. (2014). Weighted shifts and disjoint hypercyclicity. J. Oper. Theory.

[15] Kostić M. (2012). Hypercyclic and chaotic integrated *C*-cosine functions. Filomat.

[16] Salas H. (2011). Dual disjoint hypercyclic operators. J. Math. Anal. Appl..

[17] Salas H. (2013). The strong disjoint blow-up/collapse property. J. Funct. Spaces Appl..

[18] Vlachou V. (2017). Disjoint universality for families of Taylor-type operators. J. Math. Anal. Appl..

[19] Tanaka M. (2017). Property $(T_{L^{\Phi}})$ and property $(F_{L^{\Phi}})$ for Orlicz spaces $L^{\Phi}$. J. Funct. Anal..

[20] Piaggio M.C. (2017). Orlicz spaces and the large scale geometry of Heintze groups. Math. Ann..

[21] Chilin V., Litvinov S. (2017). Individual ergodic theorems in noncommutative Orlicz spaces. Positivity.

[22] Hästö P.A. (2015). The maximal operator on generalized Orlicz spaces. J. Funct. Anal..

[23] Rao M.M., Ren Z.D. (1991). Theory of Orlicz Spaces.

[24] Ansari S.I. (1997). Existence of hypercyclic operators on topological vector spaces. J. Funct. Anal..

[25] Bernal-González L. (1999). On hypercyclic operators on Banach spaces. Proc. Am. Math. Soc..

